# Tanshinone IIA promotes the apoptosis of fibroblast-like synoviocytes in rheumatoid arthritis by up-regulating lncRNA GAS5

**DOI:** 10.1042/BSR20180626

**Published:** 2018-10-05

**Authors:** Guoqing Li, Ying Liu, Fanru Meng, Zhongbin Xia, Xia Wu, Yuxuan Fang, Chunwang Zhang, Dan Liu

**Affiliations:** 1 Department of Rheumatology, Affiliated Hospital of Yangzhou University, Yangzhou City, Jiangsu Province 225000, P.R. China; 2 Clinical Medical College, Dalian Medical University, Dalian City, Liaoning Province 116044, P.R. China; 3 Department of Pathology, Northern Jiangsu People’s Hospital Affiliated to Yangzhou University, Yangzhou City, Jiangsu Province 225000, P.R. China

**Keywords:** apoptosis, GAS5, LncRNA, Rheumatoid arthritis

## Abstract

Rheumatoid arthritis (RA) is a common chronic autoimmune joint disease characteristic of elevated proliferation and infiltration of fibroblast-like synoviocytes (FLS). Here, we aimed to explore the mechanisms of the Tanshinone IIA (Tan IIA)-induced apoptosis of FLS from patients with RA (termed RAFLS). Cell Counting Kit-8 (CCK-8) assay and Annexin V staining revealed that RAFLS viability decreased and apoptosis increased after Tan IIA treatment. Long non-coding RNA (lncRNA) GAS5 expression was significantly decreased in the synovial tissues and RAFLS, while Tan IIA treatment resulted in an up-regulation of GAS5. Consistently, knockdown of GAS5 using siRNA inhibited RAFLS apoptosis. Mechanistically, GAS5 knockdown down-regulated the expression of cleaved caspase-3 and caspase-9 in the RAFLS cells and activated the phosphoinositide 3-kinase (PI3K)/AKT signaling pathway. These data indicate that Tan IIA promotes RAFLS apoptosis by up-regulating lncRNA GAS5, with enhanced expression of cleaved caspase-3/caspase-9 and inhibited PI3K/AKT signaling.

## Introduction

Rheumatoid arthritis (RA) is a chronic autoimmune joint disease characterized by proliferation and infiltration of fibroblast-like synoviocytes (FLS), which leads to synovial hyperplasia and progressive joint destruction [1]. FLS are the resident mesenchymal cells of synovial joints that produce inflammatory cytokines and chemokines. In RA, activation of FLS (termed RAFLS) and their reduced apoptosis are crucial in both the initiation and progression of arthritis [2].

One key strategy for RA treatment is to induce apoptosis of RAFLS. Tanshinone IIA (Tan IIA; 14,16-epoxy-20-nor-5(10),6,8,13,15-abietapentaene-11,12-dione) is a phytochemical derived from the roots of *Salvia miltiorrhiza Bunge* and is commonly used for treating a number of chronic diseases [3]. The therapeutic effects of Tan-IIA include anti-tumor, pro-apoptotic, and anti-inflammatory activities [4,5]. This prompted the use of Tan-IIA to attenuate proliferation of RAFLS, thus impeding the progression of RA [6].

Long non-coding RNA (lncRNA) is a novel class of non-protein coding RNAs with a length of over 200 nucleotides (nt). LncRNAs post-transcriptionally regulate gene expression by functioning as molecular sponges of other RNAs. Alterations in lncRNA expression have been discovered to underlie the activities of a plethora of drugs. In RA, the regulatory role of lncRNAs has been implicated in many studies [7,8]. It is still unclear whether lncRNAs play a role in the pro-apoptosis effects of Tan-IIA in RA. LncRNA growth arrest-specific 5 (GAS5) is a 650 nt broad-spectrum growth suppressor. GAS5 has been shown to inhibit the growth of cancers, whereby GAS5 induces apoptosis by sponging a number of cancer-related miRNAs [9]. GAS5 also exerts pro-apoptotic effects in macrophages and endothelial cells to alleviate atherosclerosis [10]. In liver fibrogenesis and oral submucus fibrosis, GAS5 inhibits fibroblasts formation by competing with miR-222, miR-21, ANRIL, etc. [11]. These evidences indicate that GAS5 is likely an important player in the anti-RA activity of Tan-IIA.

Herein, we aimed to elucidate the mechanisms of Tan-IIA in RA and the involvement of possible signaling pathways, with emphasis on the pro-apoptosis effects on RAFLS. First, we determined the viability and apoptosis of RAFLS in presence of Tan-IIA. The involvement of GAS5 in this process was explored by GAS5 knockdown. Meanwhile, we investigated the involvement of phosphoinositide 3-kinase (PI3K)/AKT signaling in the anti-RA effects of Tan-IIA as PI3K/AKT signaling is critical for the regulation of cell apoptosis. Tan-IIA has also been shown to mediate PI3K/AKT signaling in other diseases [12,13]. The protein expression of cleaved caspase-3 and caspase-9, Bax, B-cell lymphoma 2 (Bcl-2), phosphorylated (p-)P13K, P13K, p-AKT, AKT, p-mammalian target of rapamycin (mTOR), and mTOR were determined by Western blot analysis.

## Materials and methods

### Preparation of human synovial tissues and FLS

The present study was conducted in compliance to the recommendations of the Declaration of Helsinki, using protocols approved by the Medical Ethical Committee of Yangzhou University. All the participants signed informed consent. Synovial tissue samples were obtained from 16 patients with RA (9 women and 7 men, 35–74 years old) during joint replacement or synovectomy or at Northern Jiangsu People’s Hospital. Healthy synovial tissues from seven traumatic knee patients (three women and four men, 32–69 years old) were used as normal controls. Processing of synovial tissue samples were performed as described previously [12]. FLS were isolated by digestion with 2.5 g/l trypsin for 4 h at 37°C with gentle agitation.

RAFLS were cultured in Dulbecco’s Modified Eagle’s medium (DMEM, Gibco, Grand Island, NY, U.S.A.) supplemented with 10% heat-inactivated FBS (Gibco, U.S.A.), penicillin, and streptomycin. Cells from passages three to six were used in further experiments.

### Cell transfection

GAS5 siRNAs and scrambled RNAs were purchased from Dharmacon Research, Inc. (Lafayette, CO, U.S.A.). Cationic lipopolyamines (Invitrogen, Carlsbad, CA, U.S.A.) were used for RNA transfection in RAFLS at approximately 70–80% confluence. Transfection efficiency was assessed using GFP-siRNA as positive control. After 4-h incubation with transfection, medium was replaced with fresh growth medium. At 24 h after transfection, Tan-IIA (Sigma Aldrich, St Louis, MO, U.S.A.) was added to the cells and incubated for an additional 48 h.

### Real-time PCR analysis

Total RNA was extracted using the Agilent Technologies Total RNA Isolation Mini Kit (Agilent Technologies, Palo Alto, CA, U.S.A.) according to the manufacturer’s recommendations. To quantify the GAS5 expression in the TanIIA-treated RAFLS (RAFLS + Tan IIA), untreated RAFLS (RAFLS-Tan IIA), normal cells (controls) or those transfected with siRNAs (si-GAS5-1, -2, -3, or si-Scramble), real-time quantitative PCR was performed. The primers used in the present study were the following: GAS5 forward, 5′-CCCAAGGAAGGATGAG-3′ and reverse, 5′-ACCAGGAGCAGAACCA-3′; GAPDH forward, 5′-GAGTCAACGGATTTGGTCGT-3′ and reverse, 5′-TTGATTTTGGAGGGATCTC-3′. The expression level was normalized using U6 small nuclear RNA by the 2^−ΔΔ*C*^_t_ method.

### Western blot

The cultured cells were lysed in RIPA buffer, followed by centrifugation at 13000×***g*** for 15 min. Protein content in the supernatants was quantified using the BCA Protein Assay Kit (Pierce Chemical Co., Rockford, IL, U.S.A.). Twenty micrograms of protein lysates were resolved by SDS–PAGE and transferred onto nitrocellulose membranes (Millipore Corp., Bedford, MA, U.S.A.). After blocking with 5% non-fat milk for 1 h at room temperature, primary antibodies were added to the membranes. The antibodies used in the present study include rabbit polyclonal to cleaved caspase-3 (1:500, Catalog number: ab13847; Abcam, Cambridge, MA, U.S.A.), rabbit polyclonal to cleaved caspase 9 (1:500, Catalog number: ab2324; Abcam), rabbit polyclonal to Bax (1:500, Catalog number: ab10813; Abcam), rabbit polyclonal to Bcl2 (1:1000, Catalog number: ab59348; Abcam), mouse monoclonal to GAPDH (1:400, Catalog number: sc-365062; Santa Cruz Biotechnology, Inc.), rabbit polyclonal to p-PI3K (1:300, Catalog number: BS4811; Biogot Technology Co., Ltd., Nanjing, China), rabbit polyclonal to PI3K (1:1000, Catalog number: ab10813; Abcam), mouse monoclonal to Akt (1:200, Catalog number: sc-5298; Santa Cruz Biotechnology, Inc.), mouse monoclonal to p-Akt (1:800, Catalog number: ab38449; Abcam), rabbit polyclonal to mTOR (1:2000, Catalog number: ab2732; Abcam) and rabbit polyclonal to p-mTOR (1:800, Catalog number: ab1093; Abcam). Then, horseradish peroxidase (HRP)-conjugated secondary antibody (Santa Cruz Biotechnology, Inc.) was added. Images of the blots were acquired on the Fluor-S MAX MultiImager, and signal intensities were measured using Quantity One Image Software (both from Bio-Rad Laboratories, Inc., Hercules, U.S.A.).

### Cell viability assay

The Cell Counting Kit-8 (CCK-8) (Dojindo Laboratories, Kumamoto, Japan) was used to evaluate RAFLS viability in accordance with the manufacturer’s recommendations. A 100 μl suspension of RAFLS treated with Tan IIA was seeded in a 96-well plate and cultured in medium containing various concentrations (0, 1, 5, 10, 20, 40, and 80 µM) of Tan IIA. Following 24, 48, and 72 h culture, CCK-8 reagent (10 μl) was added to each well and cultured for another 1–4 h at 37°C. The optical density (OD) values of the solution were measured using a microplate reader (Varian Medical Systems, Inc., Palo Alto, CA, U.S.A.) at 450 nm.

### Analysis of apoptosis and proliferation

Annexin V-FITC Apoptosis Detection Kit I (BD Pharmingen, Heidelberg, Germany) was used to analyze RAFLS apoptosis. Following transfection with si-GAS5 or si-Scramble, the cells were harvested by trypsinization, washed with PBS, and seeded on six-well plates at the concentration of 1 × 10^6^ cells/ml. After 72 h, the cells were fixed by 70% pre-cooled ethanol and added 100 µl RNase (10 mg/ml). Five microliters of Annexin V-FITC and propidium iodide (PI) were then added to stain the cells at dark for 15 min. Apoptosis was quantified using a FACSCalibur flow cytometer and CellQuest software (both from Becton–Dickinson, Mountain View, CA, U.S.A.). The apoptosis rate was calculated as the combined percentage of cells that underwent early apoptosis (FITC^+^/PI^−^) and advanced apoptosis and necrosis (FITC^+^/PI^+^). Proliferation of cells was determined by BrdU Cell Proliferation Assay Kit (Cell signaling technology, U.S.A.).

### Statistical analysis

Student’s *t*test was used to analyze the differences between the two groups. One-way ANOVA was used to compare the results of more than two groups. Differences with a *P*-value of less than 0.05 was considered statistically significant. All statistical analyses were performed using the SAS 6.12 software package.

## Results

### Tan-IIA attenuates the viability of RAFLS

**Figure 1:**
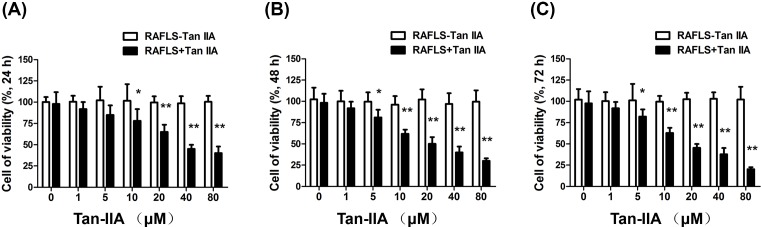
Tan-IIA attenuates the viability of RAFLS RAFLS cultured in RPMI-1640 medium with 10% FBS and treated with various concentrations of Tan-IIA for (**A**) 24 h, (**B**) 48 h, and (**C**) 72 h. Each point is the mean ± S.D. of three experiments. *P<0.05, **P<0.01, compared with untreated cells.

We hypothesized that Tan-IIA ameliorates RA by inhibiting the viability of RAFLS. To test this hypothesis, CCK-8 assay was used to assess the viability of RAFLS treated with various concentrations of Tan-IIA for 24, 48, and 72 h. Our results demonstrated that the viability of RAFLS was reduced by Tan-IIA treatment in a dose- and time-dependent manner (Figure 1A–C). At 40 μM, viability of cells was lower than 50% in all treatment durations. Therefore, 40 μM was used for further studies.

### Tan-IIA increases the apoptosis of RAFLS

**Figure 2:**
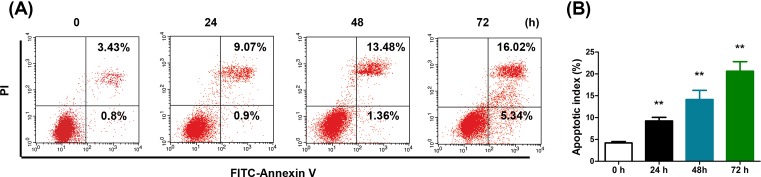
Tan-IIA increases the apoptosis of RAFLS (**A**) FACS analysis were performed to examine cell apoptosis and (**B**) apoptotic index after cells were treated with 40 μM Tan-IIA for 0, 24, 48, and 72 h. **P<0.01, compared with 0 h cells.

We next examined if Tan-IIA increased cell apoptosis of RAFLS. After treatment with Tan-IIA for 24, 48 or 72 h, cells were stained with Annexin-V-FITC/PI and FACS analysis was performed to quantify apoptotic cells. In line with the viability assay in Figure 1, increasing cell apoptosis was shown with longer incubation of Tan-IIA (Figure 2A,B). Consistently, we showed that proliferation of RAFLS was also inhibited by Tan IIA (*P*<0.01) (Supplementary Figure S1B). Together, these data validated the cytotoxicity of Tan-IIA in RAFLS.

### lncRNA GAS5 expression is down-regulated in RAFLS and Tan-IIA treatment up-regulates GAS5

**Figure 3:**
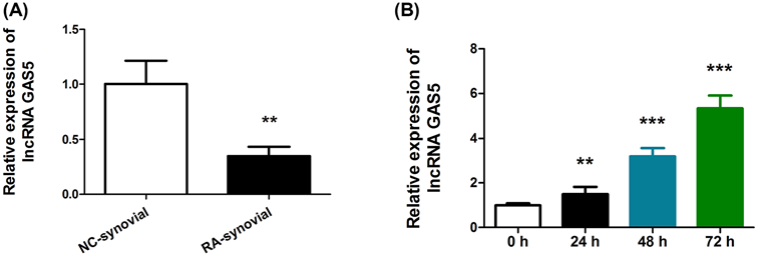
lncRNA GAS5 expression was down-regulated in the synovial tissues and up-regulated after RAFLS treatment (**A**) The expression of GAS5 was detected in synovial tissues (**A**), and (**B**) RAFLS treated synovial tissues with 40 μM Tan-IIA for 24, 48, and 72 h using qRT-PCR. The expression of GAPDH was used as an internal control to normalize GAS5 levels. **P<0.01, ***P<0.001, compared with 0 h.

To elucidate the underlying mechanism of Tan-IIA in RA, we explored the role of lncRNA GAS5 in the process. First, we showed that in RAFLS, there is a marked down-regulation of GAS5, suggesting that GAS5 down-regulation could be a pathological feature of RA. Following this, we analyzed GAS5 expression after Tan-II treatment. RAFLS were exposed to Tan-IIA for 24, 48, and 72 h. Expectedly, an increasing level of GAS5 was seen (Figure 3). These data implicated that the therapeutic effects of Tan-IIA in RAFLS may be mediated by GAS5.

### GAS5 knockdown reduces the pro-apoptosis effects of Tan-IIA in RAFLS

**Figure 4:**
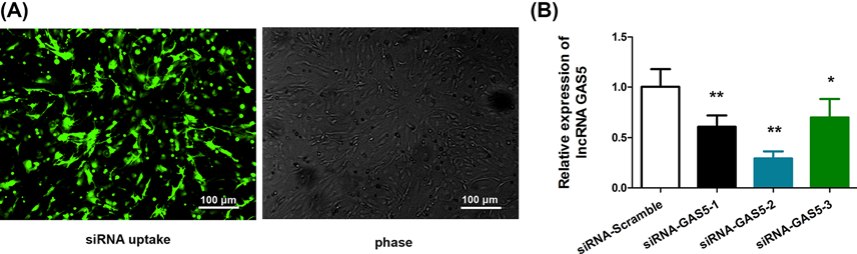
Down-regulation of GAS5 with siRNA-GAS5 (**A**) RAFLS were transfected with GFP-siRNA control as a marker of cell uptake. Fluorescent imaging (fluorescent siRNA Control) shows cells taking up the siRNA as evidenced by the green stain. Phase contrast (Phase) delineates all of the cells from the same field, and these cells do not demonstrate any obvious cytotoxicity. (**B**) GAS5 was down-regulated following transfection with siRNA-GAS5-1, -2, -3, or siRNA-Scramble for 72 h. *P<0.05, **P<0.01, compared with siRNA-Scramble.

To test the hypothesis that GAS5 is essential for the therapeutic effects of Tan-IIA in RA, we performed GAS5 knockdown, followed by evaluation of pro-apoptosis effects of Tan-IIA in RAFLS. Figure 4A,B demonstrated the uptake of GAS5 siRNA and efficiency of GAS5 knockdown in RAFLS, respectively. Three siRNAs were tested and the data showed that siRNA-GAS5-2 possessed the highest knockdown efficiency. Hence, siRNA-GAS5-2 was used in subsequent studies.

**Figure 5:**
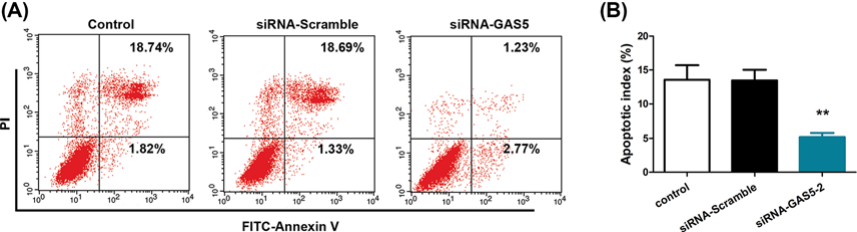
GAS5 knockdown reduces the pro-apoptosis effects of Tan-IIA in RAFLS (**A**) FACS analysis were performed to examine cell apoptosis after being treated with 40 μM Tan-IIA for 72 h. (**B**) Quantification of apoptotic index based on FACS analysis. **P <0.01, compared with control.

Consistent with our hypothesis, we showed that after siRNA-GAS5-2 transfection in Tan-IIA treated RAFLS, cell apoptosis was prominently reduced. In contrast, no apparent changes in apoptosis were seen after siRNA-Scramble transfection (Figure 5A,B). Collectively, these results confirmed that GAS5 plays a pivotal role in the pro-apoptosis effects of Tan-IIA in RAFLS.

### GAS5 knockdown inhibits the expression of caspase-3 and caspase-9, and activates the PI3K/AKT pathway

**Figure 6:**
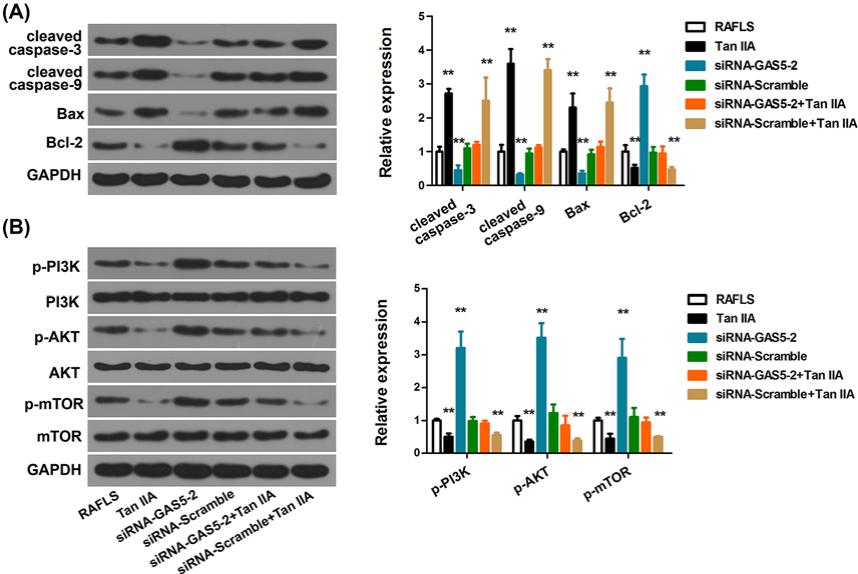
GAS5 knockdown inhibits the expression of cleaved caspase-3 and caspase-9, and activates the PI3K/AKT pathway (**A**) Western blot analyses of cleaved caspase-3 and caspase-9, Bax, and Bcl-2 protein expression in RAFLS with indicated treatments. (**B**) Western blot analyses of the PI3K/AKT pathway-related proteins in RAFLS with indicated treatments. The graph shows densitometric quantification of Western blot bands normalized to loading controls. **P <0.01, compared with RAFLS.

To unravel the precise molecular pathways involved in the Tan-IIA-induced apoptosis in RAFLS, we analyzed the expression of cleaved caspase-3 and caspase-9, Bax, Bcl-2, and PI3K/AKT pathway after GAS5 knockdown. Up-regulation of cleaved caspase-3 and caspase-9, Bax, as well as down-regulation of Bcl-2 was seen in the presence of Tan-IIA treatment, which is consistent with the decreased viability and increased apoptosis of RAFLS after Tan-IIA treatment. Transfection of siRNA-GAS5 exerted opposing effects by down-regulating cleaved caspase-3 and caspase-9, Bax, and up-regulating Bcl-2 (Figure 6A and Supplementary Figure S1A). Further, we show that Tan-IIA inactivates PI3K/Akt signaling, manifested as decreased p-PI3K, p-AKT, and p-mTOR expression (Figure 6B). Whereas, in untreated RAFLS, GAS5 knockdown substantially activated PI3K/Akt signaling and GAS5 knockdown restored the expression of p-PI3K, p-AKT, and p-mTOR. Western blot analysis of phosphorylated caspase-9 and capspase-3 (pho-caspase-9 and pho-caspase-3) indicated consistent trend (Supplementary Figure S1C,D). Altogether, these data suggested that PI3K/Akt signaling is an important underlying pathway of the regulation of Tan-IIA, in which GAS5 is a crucial mediator.

## Discussion

Clinical applications of naturally derived compounds in the management of chronic inflammatory diseases are emerging [14]. In the present study, we aimed to elucidate the mechanism of Tan-IIA in ameliorating RA, a common chronic inflammatory disease that greatly affects the morbidity and mortality of patients. The wide clinical application of Tan-IIA can be exemplified by its use as an anti-cancer drug, whereby the pro-apoptotic effects in cancer cells potently impede cancer progression [12]. Tan-IIA is also effective in alleviating inflammation induced by ischemia–reperfusion injury [13] and lipopolysaccharide [15], etc. Despite a few reports on the therapeutic effects of Tan-IIA in RA, the molecular mechanism of Tan-IIA still remains largely unclear and the interaction between Tan-IIA and RAFLS, the major component of synovial tissues associated with joint damage, has not been explored. Dysregulated RAFLS proliferation is responsible for synovial hyperplasia and the production of pro-inflammatory cytokines, which aggravates the destruction of joint. Here, we demonstrate that Tan-IIA adopts a pro-apoptotic role in RAFLS, which at least in part accounts for the beneficial effects of Tan-IIA in RA. Our result reiterated the importance of Tan-IIA as a pro-apoptotic molecule. However, we did not probe the involvement of immune cells or inflammatory cytokines in the therapeutic effects of Tan-IIA in RA. It is worth noting that the influx of innate and adaptive immune cells, including macrophages, neutrophils, T cells, and B cells into the synovial joint space, is another key factor that contributes to joint inflammation and damage in RA [16]. Neutrophils are the most abundant immune cells in synovial fluid and pannus/cartilage interface collected from the joints of RA patients. Tan-IIA has been shown to regulate neutrophil recruitment and proliferation [17]. Moreover, inflammatory cytokines secreted by RAFLS facilitate immune cell accumulation and thus join destruction [18]. Therefore, the ameliorating effects of Tan-IIA are probably attributable to the synergic effects of attenuated recruitment of RAFLS, immune cells, and decreased inflammatory cytokine expression caused by Tan-IIA. Further researches are in need to verify this hypothesis.

We further showed that lncRNA GAS5 plays an important role in the pro-apoptotic effects of Tan-IIA in RAFLS. Knockdown of GAS5 abrogated viability-reducing and apoptosis-inducing effects of Tan-IIA in RAFLS. These evidences are in agreement to recent findings that lncRNAs are pivotal regulators of RA [7,8,19]. Our study is paralleled by a number of studies demonstrating lncRNAs as important mediators of other naturally derived bioactive drugs. For example, Pan et al. [19] demonstrated that Quercetin, a flavonoid molecule, promotes apoptosis in RAFLS by up-regulating metastasis-associated lung adenocarcinoma transcript 1 (MALAT1). However, to our best knowledge, our study is the first report on the interaction of lncRNA and Tan-IIA in RA. Similar to Tan-IIA, GAS5 itself is known for inhibiting cancer growth via apoptosis-induction [20]. GAS5 was also shown to inhibit myofibroblasts activity in oral submucous fibrosis [21]. Our data implicated the potential of GAS5 as an anti-RA agent. Considering recent advances in the development of gene delivery vehicles, it is quite possible that GAS5 overexpression could be a viable tool for inhibiting RAFLS proliferation in RA.

In addition, the activation of PI3K/AKT pathway upon Tan-IIA treatment sheds light on the pro-apoptotic effect of Tan-IIA in RA. PI3K/AKT pathway plays an important role in cell cycle regulation. Moreover, the interplay between GAS5 and PI3K/AKT signaling has been demonstrated previously [20], which underscores the correlation between GAS5 up-regulation and pro-apoptosis effects of Tan-IIA. However, our result cannot rule out that the impacts on other signaling pathways, such as Erk-2 signaling [3], may also contribute to the anti-RA effects of Tan-IIA.

## Conclusion

In sum, here, we demonstrate that Tan-IIA promotes the apoptosis of RAFLS, in which GAS5 is an important mediator. Knockdown of GAS5 abrogated the pro-apoptosis efficacy of Tan-IIA. PI3K/AKT pathway plays an important role in the anti-RA effects of Tan-IIA.

## Supporting information

**supplementary Figure:** 

## Abbreviations

AKT: protein kinase B
CCK-8: Cell Counting Kit-8
FLS: fibroblast-like synoviocytes
GAS5: growth arrest-specific 5
lncRNA: long non-coding RNA
mTOR: mammalian target of rapamycin
nt: nucleotides
PI: propidium iodide
PI3K: phosphoinositide 3-kinase
qPCR: quantitative PCR
RA: rheumatoid arthritis
Tan IIA: Tanshinone IIA
