# Expression of Concern: Tanshinone IIA promotes the apoptosis of fibroblast-like synoviocytes in rheumatoid arthritis by upregulating lncRNA GAS5

**DOI:** 10.1042/BSR-2018-0626_EOC

**Published:** 2023-08-31

**Keywords:** apoptosis, GAS5, LncRNA, rheumatoid arthritis

The Editorial Office has been made aware of potential issues surrounding the scientific validity of this paper, hence has issued an Expression of Concern to notify readers.

It has been noted by a reader that there is a similarity of the Western blots with those in several other unrelated publications suspected of originating from a third party. The reader has also identified highly similar data between this publication, and that of Zhao et al. 2018 (doi: 10.3892/ijo.2018.4576) and Liang et al. 2017 (doi: 10.3892/or.2017.6049).

The authors were contacted regarding the concerns raised and provided the Editorial Office with copies of the original Western blots, however minor differences in the shape and spacing of the bands, as well as the addition/removal of background and surface features can be seen between the original submitted Figures and the raw Western blots images provided post-publication. The authors state that these differences are due to staining due to improper preservation.

