# Supplementary figures and images for: Tanshinone IIA promotes the apoptosis of fibroblast-like synoviocytes in rheumatoid arthritis by up-regulating lncRNA GAS5
Source: Biosci Rep. 2018 Oct 5;38(5):BSR20180626. doi: 10.1042/BSR20180626 (PMC6172423; doi:10.1042/BSR20180626)

**A**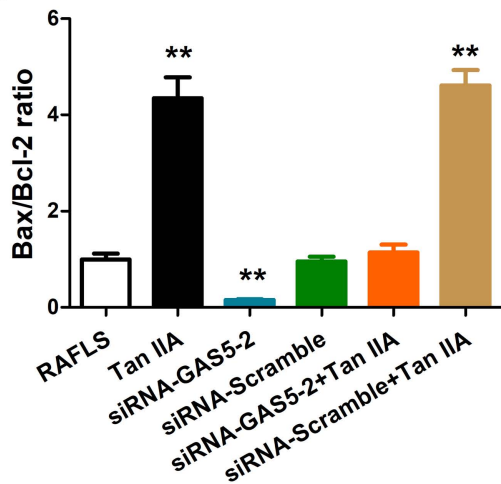**B**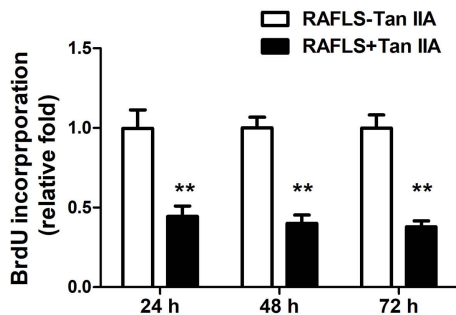**C**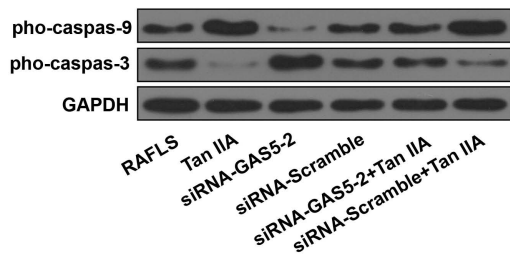**D**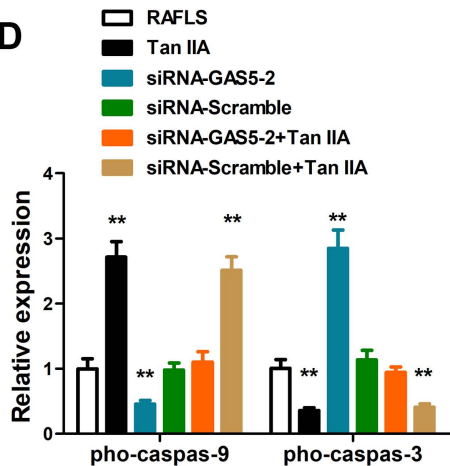

Supplement: Supplementary file 1 [file bsr20180626_Supp1.pdf]
